# Diabetes and Colorectal Cancer Risk: Clinical and Therapeutic Implications

**DOI:** 10.1155/2022/1747326

**Published:** 2022-03-07

**Authors:** Guan-Hua Yu, Shuo-Feng Li, Ran Wei, Zheng Jiang

**Affiliations:** Department of Colorectal Surgery, National Cancer Center/National Clinical Research Center for Cancer/Cancer Hospital, Chinese Academy of Medical Sciences and Peking Union Medical College, Beijing 100021, China

## Abstract

Several epidemiological studies have identified diabetes as a risk factor for colorectal cancer (CRC). The potential pathophysiological mechanisms of this association include hyperinsulinemia, insulin-like growth factor (IGF) axis, hyperglycemia, inflammation induced by adipose tissue dysfunction, gastrointestinal motility disorder, and impaired immunological surveillance. Several studies have shown that underlying diabetes adversely affects the prognosis of patients with CRC. This review explores the novel anticancer agents targeting IGF-1R and receptor for advanced glycation end products (RAGE), both of which play a vital role in diabetes-induced colorectal tumorigenesis. Inhibitors of IGF-1R and RAGE are expected to become promising therapeutic choices, particularly for CRC patients with diabetes. Furthermore, hypoglycemic therapy is associated with the incidence of CRC. Selection of appropriate hypoglycemic agents, which can reduce the risk of CRC in diabetic patients, is an unmet issue. Therefore, this review mainly summarizes the current studies concerning the connections among diabetes, hypoglycemic therapy, and CRC as well as provides a synthesis of the underlying pathophysiological mechanisms. Our synthesis provides a theoretical basis for rational use of hypoglycemic therapies and early diagnosis and treatment of diabetes-related CRC.

## 1. Introduction

Globally, colorectal cancer (CRC) is the third most common cancer in terms of incidence (10.2%) and the second leading cause of cancer-related deaths (9.0%) [1]. The number of patients with CRC is projected to reach 2.5 million worldwide by the year 2035. Most developed countries have higher rates of incidence of CRC compared to developing countries. Developing countries, including China, have also witnessed a progressive increase in incidence rates. In most cases of CRC, the time span between development of polyp and its malignant transformation to cancer, owing to inactivation of tumor suppressor genes and activation of oncogenes, is approximately 10–15 years. In addition to hereditary factors, environmental factors have also been implicated in the causation of CRC, such as obesity, excessive intake of red meat and alcohol, and smoking [2].

Type 2 diabetes mellitus (T2DM) accounts for a large proportion of disease burden among Chinese adults, and its prevalence has also shown an increasing trend [3]. T2DM and CRC have several common risk factors including obesity and sedentary lifestyle [2]. Studies investigating the association between T2DM and CRC have identified an increased risk of CRC in patients with a prior diagnosis of T2DM. There are several potential pathophysiological and molecular mechanisms linking T2DM and CRC, including hyperinsulinemia, insulin-like growth factor (IGF) axis, hyperglycemia, obesity, chronic inflammation, cytokines, adipokines, and exposure to fecal carcinogens. These underlying mechanisms may play key role in carcinogenesis and lead to worse prognosis of CRC. This review aims to summarize the epidemiological evidence and explore the potential biological mechanisms of the linkage between T2DM and CRC. Our finding provides a theoretical basis for rational use of hypoglycemic drugs and identifies potential therapeutic strategies targeting the pathophysiological mechanisms linking T2DM and CRC.

## 2. Influence of T2DM on CRC: Epidemiological Insights

### 2.1. Elevated Incidence of CRC in Patients with T2DM

Patients with T2DM are more susceptible to CRC compared with individuals with normal blood glucose level. A plethora of epidemiological studies have identified diabetes as a risk factor for CRC. Two representative large-scale prospective cohort trials are particularly noteworthy. The first epidemiological research conducted by China Kadoorie Biobank investigated more than half a million Chinese middle-aged and elderly individuals from ten distinctive areas in urban and rural regions [4]. After 10-year follow-up, diabetic patients showed an increased risk of CRC. Specifically, the hazard ratio (HR) of patients with prior diagnosis of diabetes was 1.18 [confidence interval (CI): 1.04–1.39]. Regarding the distinct anatomic sites, the risk was similar for both colon and rectal cancers. However, the linkage between diabetes and CRC was slightly more pronounced in males compared to females. The association became less apparent with increase in the duration of follow-up and was nonsignificant in patients who had been diagnosed with diabetes a decade ago. This phenomenon may potentially be attributable to the conversion from hyperinsulinemia to hypoinsulinemia due to exhaustion of pancreatic *β*-cells with progression of T2DM. Furthermore, researchers also carried out same follow-up studies in individuals without a prior diagnosis of diabetes. The results showed a positive correlation between random plasma glucose level and increased incidence of CRC, and this association appeared stronger in men, although the difference was not statistically significant. The second large-scale prospective cohort study consisted of the nurses' health study recruiting 1,217,000 female American nurses and the health professionals' follow-up study recruiting 51,529 male health professionals, respectively [5]. After 26-year follow-up, similarly, it was found that the HR between T2DM and risk of CRC was 1.42 (95% CI: 1.21–1.81) in male health professionals. However, this association between T2DM and CRC was less strong in females (HR: 1.17; 95% CI: 0.98–1.39). Given that observational studies have demonstrated a negative linkage between estrogen and incidence of CRC, sexual hormones including estrogen may play a role in the distinct risk of CRC associated with T2DM in both men and women by increasing the sensitivity of peripheral target tissues to insulin. Besides, more epidemiological studies [6–9] concerning the linkage between T2DM and CRC are shown in Table 1.

In summary, several epidemiological studies suggest a moderately increased risk of CRC in patients with T2DM. Further in-depth studies are required to exclude the influence of potential confounding factors and investigate all aspects of this association.

### 2.2. Worse Prognosis of CRC in Patients with T2DM

In addition to being a risk factor for CRC, T2DM has also been shown to exert a negative influence on the prognosis of CRC patients. One meta-analysis found that CRC patients with preexisting diabetes had higher all-cause mortality and cancer-specific mortality than CRC patients without diabetes [10]. Besides, other epidemiological studies revealed that CRC patients with preexisting diabetes witnessed higher overall mortality compared with CRC patients without diabetes [11–14]. However, the adverse effects of T2DM on the prognosis of CRC patients varied according to the anatomic site of CRC. In one study, diabetes was associated with decreased overall survival of patients with proximal colon cancer, but this negative correlation between diabetes and overall survival was not observed in patients with distal colon cancer or rectal cancer [15]. The differential impact of diabetes on the overall survival of CRC patients may be linked to the distinct biological features and therapeutic strategies for proximal and distal colon cancer and rectal cancer. Furthermore, in another study, T2DM was associated with worse recurrence-free survival of CRC patients, and many of these patients died of tumor recurrence [16]. To summarize, T2DM has been shown to adversely affect the prognosis of CRC patients; however, the underlying mechanisms of this association are not well characterized. Some studies have suggested that T2DM can stimulate liver metastasis and induce lymphatic metastasis of CRC, which may severely affect the prognosis and survival of CRC patients with preexisting diabetes.

## 3. Pathophysiological Links between T2DM and CRC

### 3.1. Hyperinsulinemia and Insulin-Like Growth Factors Axis

**Figure 1:**
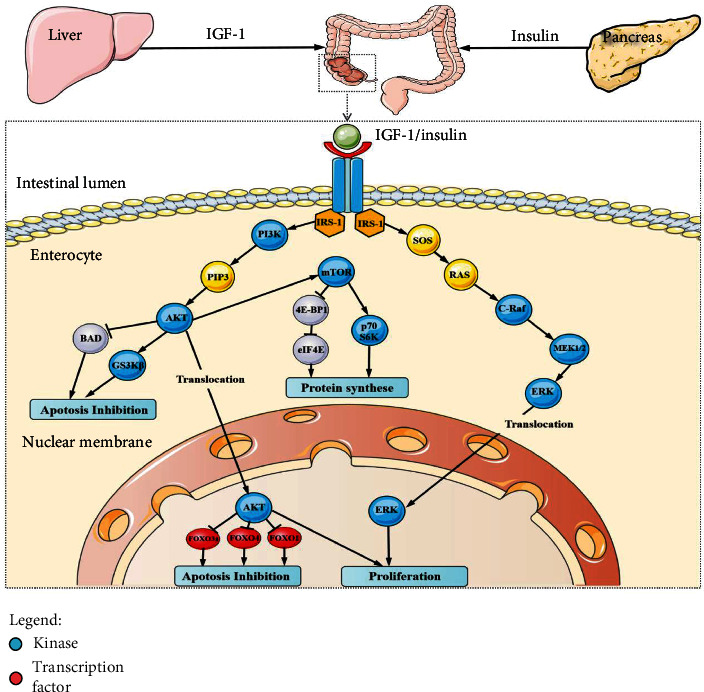
Schematic review of the tumor-promoting signaling pathways linking hyperinsulinemia and CRC. IGF-1R is comprised of extracellular *α*-chains, transmembrane *β*-chains and intracellular tyrosine kinase (IRS-1). Both IGF-1 and insulin are ligands for IGF-1R, and their binding induces autophosphorylation and conformational change of cytoplasmic tyrosine domain, resulting in stimulation of signaling cascades, mainly including PI3K/AKT and MAPK pathways which are closely correlated with protein synthesis, survival, and proliferation. IRS-1: insulin receptor substrate-1; PI3K: phosphatidylinositol 3-kinase; AKT: protein kinase B; mTOR: mammalian target of rapamycin; MAPK: mitogen-activated protein kinase; MEK: mitogen-activated protein kinase; ERK: extracellular signal-regulated kinase; SOS: son of sevenless; FOXO: Forkhead; BAD: proapoptotic member of the Bcl-family; GS3K*β*: glycogen synthase kinase.

Owing to insulin resistance, hyperinsulinemia is a compensatory response to maintain normal blood glucose level. As mentioned above, epidemiological studies have demonstrated a positive association of CRC and adenoma with T2DM, but this association has not been observed in the context of T1DM or gestational diabetes [17]. Therefore, hyperinsulinemia may play a significant role in colorectal carcinogenesis. Insulin/insulin-like growth factor (IGF) system consists of three kinds of ligands (insulin, IGF-1, and IGF-2), four types of receptors (insulin receptor, IGF-1 receptor, hybrid IGF-1/insulin receptor, and IGF-2 receptor), IGF binding proteins (IGFBP1–IGFBP6), IGFBP protease, and insulin receptor substrate (IRS1–6). Due to structural similarities among insulin receptor, IGF-1 receptor, and hybrid IGF-1/insulin receptor, insulin was shown to bind with insulin receptor, IGF-1R, and possibly hybrid IGF-1/insulin receptor [18, 19] and result in activation of intracellular signal pathways including phosphatidylinositol 3-kinase (PI3K)/mammalian target of rapamycin (mTOR) and mitogen-activated protein kinase (MAPK)/extracellular signal-regulated kinase (ERK) pathways, leading to promotion of cell proliferation and inhibition of apoptosis of cancerous colon epithelial cells [20, 21]. Specifically, the PI3K/mTOR pathway leads to cancer cell survival and migration, whereas the MAPK/ERK pathway promotes cancer cell metabolism and proliferation. The signaling pathways involved in hyperinsulinemia-induced colorectal tumorigenesis are exhibited in Figure 1.

**Figure 2:**
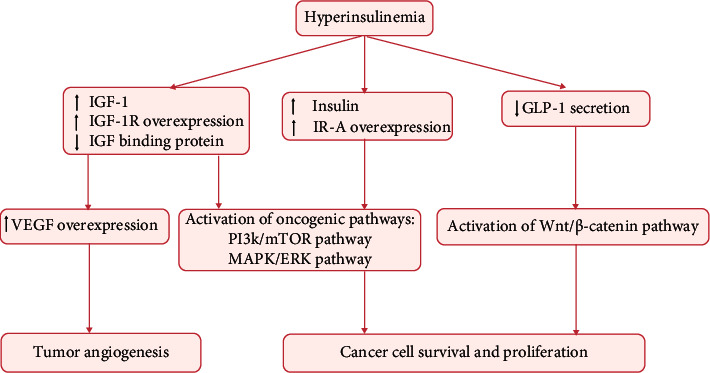
Effects of hyperinsulinemia and IGF axis on colorectal carcinogenesis. GLP-1: glucagon-like peptide-1; PI3K: phosphatidylinositol 3-kinase; mTOR: mammalian target of rapamycin; MAPK: mitogen-activated protein kinase; MEK: mitogen-activated protein kinase; ERK: extracellular signal-regulated kinase.

Insulin injection has been shown to induce carcinogenesis in normal colorectal epithelial cells [22]. Moreover, high level of insulin stimulates the production of IGF-1 by liver cells after binding of insulin with insulin receptor. Similarly, IGF-1 is regarded as a mitogen and can bind with insulin receptor and IGF-1 receptor owing to the structural homology between insulin receptor and IGF-1 receptor. The subsequent downstream intracellular signal transduction cascades induced by IGF-1 and insulin are identical. IGF-1 is capable of promoting cell growth and inhibiting apoptosis [23, 24] through the PI3K/mTOR and MAPK/ERK pathways. Apart from the direct mitogenic effects, hyperinsulinemia may also elevate the bioactivity of IGF-1 by decreasing the expression of IGFBPs [25]. Besides, IGF-1 can facilitate tumor angiogenesis by upregulating the expression of vascular endothelial growth factor (VEGF), thereby contributing to the growth and metastases of colorectal tumor [26, 27]. Additionally, IGF-1 has the ability to alter integrins and regulate the action of the E-cadherin-*β*-catenin complex, promoting migration of colon epithelial tumor cells [28]. IGF-1 is a potent promoter of CRC, which is consistent with the findings of epidemiological studies wherein patients with acromegaly with high serum level of IGF-1 exhibited greater susceptibility to CRC [29–31]. Hence, apart from CEA, CA19-9, and other commonly used biomarkers of CRC, serum level of IGF-1 and the ratio of IGF-1 to IGFBP3 are potential novel tumor makers with diagnostic and prognostic relevance in patients with coexisting CRC and T2DM. Another molecular mechanism linking hyperinsulinemia and CRC involves glucagon-like peptide-1 (GLP-1). Insulin resistance decreases the secretion of GLP-1, leading to stimulation of Wnt/*β*-catenin pathway and overexpression of oncogenes including c-Myc and cyclin D1, which can induce tumor cell proliferation and survival [32]. The tumor-promoting effects induced by hyperinsulinemia are shown in Figure 2.

### 3.2. Hyperglycemia

**Figure 3:**
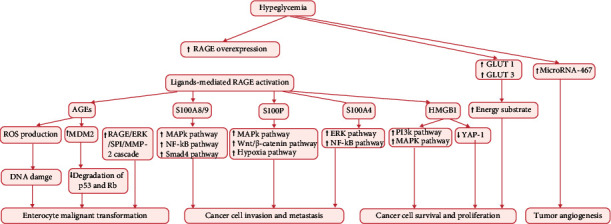
Effects of hyperglycemia on colorectal carcinogenesis. RAGE: receptor for advanced glycation end products; AGEs: advanced glycation end products; ROS: reactive oxygen species; ERK: extracellular signal-regulated kinase; SP1: specificity protein 1; MMP-2: matrix metallopeptidase-2; MAPK: mitogen-activated protein kinase; NF-*κ*B: nuclear factor kappa-B; PI3K: phosphatidylinositol 3-kinase; YAP-1: Yes-associated protein-1; GLUT1: glucose transporter 1; GLUT3: glucose transporter 3.

Glucose is an essential source of energy for cell growth and proliferation, while hyperglycemia of diabetes provides an ideal milieu for survival of tumor cells. According to the special metabolic phenomenon referred to as the “Warburg effect” [33], tumor cells prefer obtaining energy by glycolysis instead of mitochondrial oxidative phosphorylation. Therefore, chronic hyperglycemia may stimulate the proliferation of tumor cells by directly fueling them. Additionally, within the hypoxic tumor microenvironment, the expressions of glucose transporter (GLUT), including GLUT 1 and GLUT 3, are upregulated by mutations in *Akt* oncogene [34] and *K-RAS* [35] oncogene, promoting the utilization of glucose as the major energy substrate by the tumor cells. Moreover, hyperglycemia can cause accumulation of advanced glycation end products (AGEs) [36], leading to stimulation of chronic inflammation, oxidative stress, and tumorigenesis. The expression of AGEs receptor (RAGE), which is found to be associated with survival of colon cancer cells [37], is upregulated owing to the higher concentration of AGEs [38]. Thus, enhanced AGEs-RAGE interaction may be the missing link between diabetes and increased risk of CRC [39]. The interaction between RAGE and AGEs induces recruitment and activation of inflammatory cells releasing inflammatory cytokines, which are involved in many cancer-related signal pathways [40]. Moreover, the expressions of AGEs and RAGE were found to be upregulated in colonic mucosa of azoxymethane-injected mice fed with high-glucose diet. This hyperglycemia-related upregulation of AGEs and RAGE stimulated continuous production of reactive oxygen species, which can cause DNA damage and contribute to malignant transformation of colonic epithelial cells. Besides, elevated expression of AGEs and RAGE showed a correlation with increased colon adenomas, and this tumor-promoting effect was partially attenuated by AGE inhibitory agents including metformin and losartan [41]. In addition, one study found increased serum levels of AGEs in CRC patients, which contributed to invasion and metastasis of CRC by activating RAGE/ERK/specificity protein 1 (SP1)/matrix metallopeptidase-2 (MMP2) cascade in cancerous tissue; moreover, the RAGE blocking antibody and SP-1 siRNA was found to reverse the AGE-induced effects [42]. Besides, one study indicated that diabetes elevates the level of AGEs, which can upregulate the expression of MDM2 via transcription factor KLF5 in colon cells. MDM2 could bind with tumor suppresser genes including *p53* and *Rb*, resulting in degradation of p53 and Rb [43]. Furthermore, intensive studies have indicated that high-glucose diets induce AGEs-RAGE interaction, promoting the initiation and progression of CRC [41, 44]. Besides AGEs, RAGE can also bind with other ligands, such as high mobility group box 1 (HMGB1), S100A8/9, S100A4, and S100P. HMGB1 is one of the ligands of RAGE, and HMGB1-RAGE signaling plays a role in colon carcinogenesis. In a study, HMGB1-mediated RAGE activation was shown to reduce the accumulation of transcription factor Yes-associated protein-1 (Yap-1) in colon tumor cells in a RAS-dependent manner. Through modulating Yap-1 and RAS pathway, HMGB1-RAGE signaling can drive stemness and development of CRC [45]. Furthermore, interaction of HMGB1 and RAGE prompts colonic myofibroblast proliferation, migration, and invasion through the PI3K and MAPK pathways [46]. In another study, activation of RAGE by HMGB1 was found to stimulate ERK1/2, leading to phosphorylation of mitochondrial dynamin-related protein 1 (Drp1) and chemoresistance in CRC [47]. S100A8/9 are important ligands for RAGE in the process of carcinogenesis in CRC. The interaction of S100A8/9 and RAGE promotes tumor migration, invasion, and metastasis by triggering different signaling pathways, including MAPK, Smad4, and NF-*κ*B signaling pathways [48–52]. As another vital member of the S100 family, the calcium-binding protein S100A4 is the ligand of RAGE. The interaction of S100A4 with RAGE was found to increase CRC cell motility by activating MAPK/ERK, Wnt/*β*-catenin, and hypoxia signaling pathways [53, 54]. In addition, S100P was shown to be overexpressed in CRC cells and to promote epithelial-mesenchymal transition and metastasis of CRC through interaction with RAGE followed by activation of ERK and NF-*κ*B pathway [55, 56]. In addition, activation of RAGE was also shown to upregulate the expression of VEGF [57], while hyperglycemia was found to induce tumor angiogenesis through upregulating microRNA-467, which is a suppressor of antiangiogenic protein thrombospondin-1 [58]. However, it is difficult to validate the independent effect of hyperglycemia in the development of CRC since hyperinsulinemia and hyperglycemia coexist in most patients with diabetes. The streptozotocin-induced hyperglycemia animal model has been used to study the influence of hyperglycemia on tumor growth without concomitant hyperinsulinemia. It showed that hyperglycemia inhibits the growth of human prostate, breast, and pancreatic cancer cells in the absence of hyperinsulinemia [59]. Biological links between hyperglycemia and CRC are integrated in Figure 3. However, no study has investigated whether hyperglycemia is an independent tumor-promoting factor for CRC.

### 3.3. Adipose Tissue Dysfunction and Inflammation

**Figure 4:**
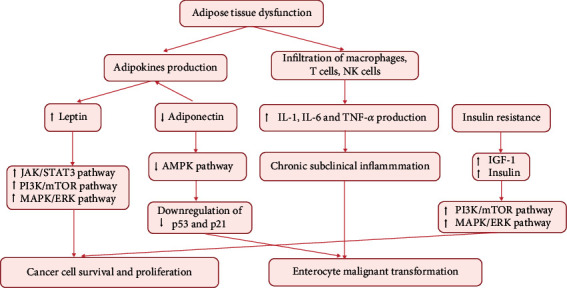
Effects of obesity and adipose tissue dysfunction on colorectal carcinogenesis. JAK: Janus kinase; STAT3: signal transducer and activator of transcription 3; PI3K: phosphatidylinositol 3-kinase; mTOR: mammalian target of rapamycin; MAPK: mitogen-activated protein kinase; ERK: extracellular signal-regulated kinase; AMPK: adenosine monophosphate-activated protein kinase; IL-1*β*: interleukin-1*β*; IL-6: interleukin-6; TNF-*α*: tumor necrosis factor-*α*.

Approximately 80%–90% patients with T2DM have concomitant obesity [60]. Diabetes and obesity interact mutually: obesity-induced inflammatory factors can impair pancreatic *β*-cells, while chronic hyperinsulinemia and hyperglycemia in turn lead to visceral adiposity [61]. Obesity is correlated with dysfunction of adipose tissue which is characterized by chronic inflammation [62]. Adipose tissue can act as an endocrine organ and actively produce inflammatory factors, free fatty acids, adipokines, proangiogenic factors, and extracellular matrix components, ultimately contributing to construction of tumor-supporting microenvironment. Due to insufficient formation of the blood vessels in the setting of obesity, adipose tissue may be in a hypoxic state, which induces the infiltration of macrophages, T cells, natural killer (NK) cells, and other inflammatory cells. These inflammatory cells can produce a variety of proinflammatory cytokines including IL-1*β*, IL-6, and TNF-*α*, which play an important role in the etiopathogenesis of tumors [63]. The chronic inflammation creates a tumor-supporting microenvironment that is in favor of tumor angiogenesis [64]. Moreover, adipose-induced production of these proinflammatory cytokines induces insulin resistance and enhances the tumor-promoting effects of hyperinsulinemia [65]. Furthermore, adipose tissue can produce adipokines including leptin and adiponectin, which exhibit a strong correlation with CRC. Accumulation of visceral adipose tissue promotes leptin production and represses adiponectin production. Studies have demonstrated mitogenic and antiapoptotic effects of leptin [66]. The interaction of leptin and its receptor (Obr) can promote tumor growth and metastases by activating several intracellular signaling pathways including JAK/STAT3, MAPK, and PI3K/Akt pathways. Leptin has been shown to enhance the invasiveness of colonic tumor cells [67]. In particular, the overexpression of leptin was shown to correlate with CRC progression [68] and poor prognosis [69]. On the contrary, adiponectin exhibits protective effects against colorectal carcinogenesis mediated via activation of its receptors (ADIPOR1 and ADIPOR2). The interaction of adiponectin and its receptors activates adenosine monophosphate-activated protein kinase (AMPK) pathway, which induces the activation of tumor suppressor genes *p53* and *p21*, subsequently leading to cell apoptosis and proliferation inhibition [70]. Adiponectin-induced activation of the AMPK pathway was shown to downregulate the PI3K/Akt pathway, which plays a vital role in the process of colorectal tumorigenesis promoted by diabetes [71]. In addition, obesity can have a deleterious effect on the intestinal microbiota by increasing intestinal permeability, leading to entry of lipopolysaccharides (LPS) produced by gram-negative bacteria in the gut into the abdominal cavity and causing low-grade inflammation favoring malignant transformation [72, 73]. The pathophysiological mechanisms between obesity and CRC are shown in Figure 4.

### 3.4. Gastrointestinal Motility Disorder

Intestinal autonomic nervous dysfunction is a common complication of diabetes, occurring in the majority of patients with advanced T2DM. Longer bowel transit time caused by diabetic neuropathy and increased exposure level of fecal carcinogens is another potential pathogenic mechanism linking T2DM and CRC. Several carcinogens including bile acids, ammonium, and fecapentaene-12 in the stool may promote carcinogenesis of colonic mucosa [74]. Moreover, high levels of fecal bile acids were found in patients with T2DM [75] which have been shown to promote colorectal cancer development [76–78]. Secondary bile acids in the gut, mainly including deoxycholic acid (DCA) and lithocholic acid (LCA), may impair the structural and functional integrity of colonic cells [79].

### 3.5. Impaired Immunological Surveillance

**Figure 5:**
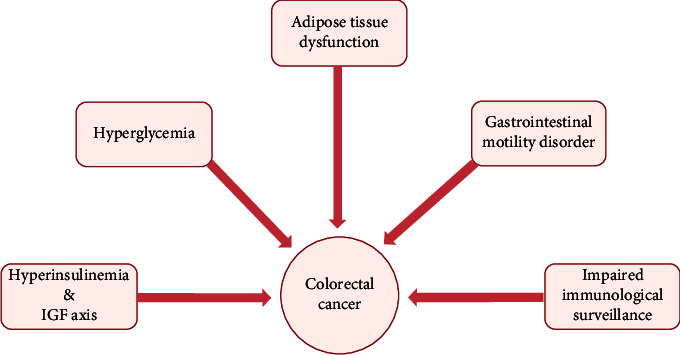
Predisposing factors of patients with T2DM for CRC.

Tumor cells can be identified and eliminated by the immune system under normal conditions. Cellular immunity plays a predominant role in tumor-related immunological surveillance. CD4^+^ T cells are critical in cellular immunity, and the ratio of CD4^+^ T cells to CD8^+^ T cells is a marker of function of the immune system. Impaired cellular immunity is indicated by decrease in the ratio of CD4^+^ T cells to CD8^+^ T cells. One study found reduced level of CD4^+^ T cells along with decreased ratio of CD4^+^ T cells to CD8^+^ T cells in T2DM, which indicates that progression of T2DM may impair the immune system, particularly cellular immunity. This is one of the mechanisms of the increased susceptibility of diabetic patients to cancer [80]. Additionally, NK cells also participate in antitumor immune response by recognizing and killing tumor cells. Another study found reduced activity of NK cells in patients with diabetes and CRC, leading to decline in antitumor capacity of NK cells [80]. Besides, disordered metabolism of vitamin C caused by hyperglycemia may be detrimental to immune system.  Figure 5 represents the main predisposing factors linking T2DM and CRC.

## 4. Novel Anticancer Agents Targeting Vital Receptors in T2DM-Induced Colorectal Tumorigenesis

Hyperinsulinemia and hyperglycemia are the most prominent pathophysiological features that play a key role in T2DM-induced colonic carcinogenesis through activating IGF-1R and RAGE, respectively. Therefore, IGF-1R and RAGE are potential therapeutic targets for CRC, especially for CRC patients with T2DM. IGF-1R and RAGE inhibitors are promising anticancer agents for CRC patients with T2DM. In this section, we discuss the findings of preclinical and clinical studies of anticancer agents targeting IGF-1R and RAGE.

### 4.1. Anticancer Agents Targeting IGF-1R

As previously mentioned, insulin/IGF axis plays a vital role in the development and progression of CRC. Within the insulin/IGF axis, IGF-1R is capable of stimulating the proliferation and migration of tumor cells through binding with insulin and IGF-1 and activating the downstream tumor-promoting signal pathways. Moreover, the expression of IGF-1R in CRC tissues was shown to be significantly higher than that in normal colon tissues and was associated with more advanced stage and worse prognosis [81]. Therefore, IGF-1R may be considered as a potential therapeutic target, especially for CRC patients with T2DM. IGF-1R consists of three components, two *α*-chains located outside the cell which can bind with its ligands, two transmembrane *β*-chains, and one intracellular tyrosine kinase, which can be phosphorylated through combination of *α*-chains and ligands, resulting in activation of the PI3K/mTOR and MAPK/ERK signal pathways. Accordingly, IGF-1R targeting agents can be divided into three categories: (1) monoclonal anti-IGF1R antibodies, which block the binding of IGF-1R and its ligands and induce internalization of IGF-1R; (2) small-molecule TKIs which are involved in restraining autophosphorylation of intracellular tyrosine kinase through competitively binding with ATP, which is supposed to bind to the IGF-1R kinase domain; and (3) IGF ligand antibodies. Some preclinical studies of IGF-1R inhibitory agents have shown promising results. For instance, MK-0646, a type of monoclonal anti-IGF1R antibody, was found to induce apoptosis of colon cancer cells and inhibit growth of CRC cells in mice; the volume and weight of tumor xenografts were dramatically reduced in mice treated with MK-0646. This study also demonstrated that a TKI called OSI-906 could constrain the proliferation and stimulate apoptosis of CRC cells *in vitro*, consequently leading to decreased growth of xenograft tumors [82]. Additionally, another potent monoclonal anti-IGF1R antibody called CP751,871 (also termed as figitumumab) showed anticancer activity as well. In an animal study, CP751,871 was found to restrain the growth of xenograft tumors derived from the colo-205 cell line. Besides, in this study, concomitant treatment with CP751,871 substantially improved the anticancer efficacy of other standard chemotherapeutic agents [83]. In another study, the IGF-1R/insulin receptor TKIs PQIP exhibited increased antitumor effects when administered in combination with chemotherapeutic drugs (including oxaliplatin, irinotecan, and 5-fluorocrail) against CRC xenografts derived from HT-29, LS513, and HCT116 cell lines [84]. These preclinical studies suggest that blockade of IGF-1R is a potential therapeutic strategy against CRC, especially for diabetic patients who have higher level of IGF-1R ligands, including insulin and IGF-1. However, the outcomes of clinical trials of IGF-1R blockade have been far from satisfactory. The monoclonal anti-IGF1R antibody CP751,871, which potent antitumor activity in animal experiments [83], conferred no survival advantage in patients with refractory metastatic CRC. None of the 168 patients enrolled in this clinical trial achieved objective partial or complete response after receiving intravenous therapy of CP751,871 [85]. In another clinical trial, monoclonal anti-IGF1R antibody SCH71745 also failed to improve the prognosis of patients with advanced CRC [61]. In addition to unsatisfactory outcomes of single IGF-1R inhibitory agents in several clinical trials, a combination of monoclonal anti-IGF1R antibody MK-0646 [86] and IMC-A12 [87] with epidermal growth factor receptor inhibitor cetuximab was not found to improve the prognosis of patients with advanced CRC. Moreover, in a randomized placebo-controlled clinical study, few patients with advanced CRC who were refractory to first-line chemotherapy gained survival benefits from combination of monoclonal anti-IGF1R antibody AMG 479 (ganitumab) and FOLFIRI (tetrahydrofolic acid, irinotecan, and 5-fluorouracil) chemotherapy in terms of median progression free survival [88]. However, clinical trials focusing on the efficacy of IGF-1R-targeted agents in patients with CRC have shown disappointing results. Moreover, IGF-1R blockade may result in many adverse effects, such as hyperglycemia and impaired glucose tolerance, which cannot be ignored in CRC patients with T2DM. Based on failure of clinical trials investigating the efficacy of IGF-1R inhibitory agents for patients with CRC, application of IGF-1R blockage in unselective CRC population is not recommended. Moreover, despite the important role of IGF system in the carcinogenesis and progression of CRC, the expression and oncogenic activity of IGF-1R and its ligands may vary according to the anatomic location of the primary tumor and molecular profiles. Studies have found higher expressions of several components of the IGF system (including IGF-1, IGF-2, and IGF-1R) in normal rectal mucosa compared with colonic mucosa [89], which probably suggests a more pronounced tumor-promoting effect of the IGF system in rectal cancer. Therefore, in view of the heterogenous biological features and molecular subsets in CRC, more investigations of IGF-1R-targeted agents in selected CRC population based on molecular biomarkers are warranted. Methods to identify specific subsets of CRC patients who are more sensitive to IGF-1R-targeted agents are key challenges that must be addressed.

### 4.2. Anticancer Agents Targeting RAGE

As noted above, several tumor-related signaling pathways induced by multiligand activation of RAGE play a vital role in the proliferation and invasion of CRC cells in the context of hyperglycemia. Thus, RAGE may be a potential therapeutic target for CRC patients, especially those with concomitant diabetes. There have been intensive studies concerning inhibitory agents against RAGE multiligand including AGEs, HMGB1, and S100A9. Fluoroquinolones were found to inhibit AGEs by increasing the scavengers of precursor of AGEs and showed unselective cytotoxicity against CRC cell lines, such as HT29, HCT116, SW620, CACO2, and SW480 [90]. Besides, flavonoids and polyphenolic acids extracted from *Castanea mollissima* Blume (also called Chinese chestnut) showed stronger inhibitory effects on AGEs and cytotoxic activity against the human COLO 320 DM colon cancer cells [91]. Another extract from *Carpobrotus edulis* in South Africa surprisingly repressed the production of AGEs. Incubation of HCT116 cells with ethanol-water extracts of *Carpobrotus* for 24 hours significantly decreased the cell viability [92]. Moreover, protocatechuic acid and 3,4-dihydroxyphenylacetic acid (DHPA) inhibited the formation of AGEs [93]; however, whether protocatechuic acid and DHPA can restrain proliferation of human CRC cell lines needs further validation. Apart from AGEs, S100A9 is the ligand for RAGE and plays a role in invasive growth of CRC. The anti-S100a9 antibody was shown to repress tumor cell proliferation and inflammatory response in the colonic tumor tissue of mice. Gene expression profiling revealed that treatment of mice with anti-S100a9 antibody suppressed vital signaling pathways involved in CRC including the PI3K-Akt and Wnt signaling pathways [94]. As for HMGB1, it was found that shRNA [95] and antisense oligonucleotides [44] targeting *HMGB1* gene can inhibit the proliferation and migration of colonic tumor cells. Currently, there is a paucity of studies that have focused on RAGE inhibitory agents and most of these studies were preclinical studies. There is still a long way to go from preclinical experiments to clinical practice.  Table 2 summarizes the preclinical and clinical studies concerning anticancer agents targeting IGF-1R and RAGE against CRC.

## 5. Effects of Hypoglycemic Therapies on CRC: Therapeutic Implications

Effective management of blood glucose is of vital importance for diabetic patients. Initially, most patients can achieve glycemic control with oral hypoglycemic drugs. However, with progression of T2DM, many patients have to inevitably accept insulin therapy. According to the different pharmacological effects on endogenous insulin, oral hypoglycemic drugs can be divided into two main categories: insulin-sensitizing drugs (including metformin and thiazolidinedione) and insulin-secreting drugs (including sulfonylureas). Different types of oral hypoglycemic drugs exert distinct effects on the incidence and prognosis of CRC in diabetic patients. Metformin use is associated with reduced risk of CRC in T2DM patients and improved prognosis of CRC patients with preexisting T2DM. Another insulin sensitizer thiazolidinedione was found to protect diabetic patients against CRC. As for insulin-secreting drugs, the use of sulfonylureas was associated with increased incidence of CRC in patients with T2DM. Exogenous insulin therapy, which is unavoidable in many patients to achieve glycemic control, places diabetic patients at a higher risk of CRC. Therefore, further studies are required to inform selection of appropriate therapeutic strategies and choice of hypoglycemic drugs to reduce the risk of CRC in diabetic patients.  Table 3 provides a nonexhaustive list of association studies between hypoglycemic medications and CRC.

### 5.1. Insulin and Its Analogs

Insulin is indispensable and inevitable for blood glucose management of patients who present in the late stage of T2DM. As mentioned before, insulin is regarded as strong growth factor and mitogen which is able to stimulate cell proliferation and inhibit apoptosis and increase bioactivity of IGF-1 to exert tumor-promoting effects. It was demonstrated by animal trials that exogenous injection of insulin could facilitate colon tumor growth in mice. Cohort and nested case-control studies also revealed that elevated level of C peptide was positively associated with risk of CRC [96, 97]. Therefore, long-term exogenous use of insulin may increase level of insulin of diabetic patients, increasing risk of CRC consequently. Accordingly, several meta-analyses and cohort studies did demonstrate that the use of exogenous insulin was correlated with increased risk of CRC [98–102]. Besides, other relevant studies pointed that the use of insulin could worsen outcomes of CRC patients with preexisting diabetes, in which the use of insulin was associated with more lymphatic metastasis and more advanced pathological stage. In summary, exogenous insulin may not only increase risk of CRC in diabetic patients but also has negative impact on prognosis of diabetic patients who have already been diagnosed with CRC. Meanwhile, studies found that the use of long-acting insulin analogs (insulin glargine and detemir) did not increase risk of CRC for patients with T2DM [103, 104], but at the same times, one cohort study found no evidence of significant differences in risk for ten cancers including CRC for insulin glargine or insulin detemir use compared with human insulin [105]. Given the increased incidence of CRC caused by hyperinsulinemia, whether insulin analogs could become hopeful alternative to insulin therapy still urgently needs more clinical trials for further verification.

### 5.2. Metformin

As an important insulin-sensitizing agent, metformin, which could relief insulin resistance and reduce the tumor-promoting effect of hyperinsulinemia, is widely used for diabetic patients. Moreover, insulin is also capable of downregulating AMPK pathway and upregulating mTOR pathway to inhibit metabolism and mitosis of tumor cells and selectively killing tumor stem cell [106]. Multiple meta-analyses found that metformin use could decrease incidence of CRC [107–109]. And another cohort study also found that the medication of metformin resulted in lower incidence of CRC in diabetic patients to normal level [110]. Besides, a clinical trial indicated that diabetic patients who accepted joint use of metformin and FOLFOX6 chemotherapy witnessed better therapeutic efficacy and higher 5-year survival rate than these patients who merely accepted FOLFOX6 chemotherapy. Similarly, other studies found that the use of metformin was positively correlated with better overall survival and lower postoperative morbidity in patients with CRC and preexisting diabetes [111, 112]. To sum up, metformin is not only tightly associated with decreased risk of CRC in diabetic patients but also can improve prognosis of CRC patients with diabetes. Therefore, metformin can be considered as ideal antidiabetic drugs especially for patients with CRC and T2DM.

### 5.3. Thiazolidinedione

Thiazolidinedione is another critical oral antidiabetic drugs which could activate peroxisome proliferator-activated receptor-*γ* (PPAR*γ*) to increase sensitivity of peripheral target tissues to insulin and induce redistribution of adipose tissue. PPAR*γ* is highly expressed in both normal intestinal epithelial cells and colorectal cancer cells [113], and PPAR*γ* agonist including thiazolidinedione is demonstrated to be capable of inducing cell apoptosis and differentiation [114] and inhibiting carcinogenesis of normal colonic cells and colon aberrant crypt foci as well [115]. However, the results of clinical trials on thiazolidinedione are inconsistent. One case-control study recruiting 606583 patients with T2DM found that rosiglitazone was associated with reduced risk of CRC compared with those diabetic patients who had never accept rosiglitazone therapy, but this protective benefits has not seen in pioglitazone [114]. Additionally, another case-control study [115] and meta-analyses [113, 116] also found that the use of thiazolidinedione could reduce risk of CRC, whereas there are still other relevant epidemiological studies failed to conclude that thiazolidinedione could protect diabetic patients from elevated risk of CRC [108, 117]. Thus, the correlation between thiazolidinedione therapy and risk of CRC still needs more clinical studies for further validation.

### 5.4. Sulfonylurea and *α*-Glucosidase Inhibitor

Sulfonylurea, which is different from insulin sensitizer including metformin and thiazolidinedione, is another kind of oral antidiabetic drug which could decrease blood glucose by stimulating secretion of endogenous insulin. To date, researches which focus on the relationship between sulfonylurea and incidence of CRC on diabetic patients are limited, and the conclusions of these researches are incompatible. One case-control study found that the use of sulfonylurea might increase risk of CRC in diabetic patients who were elder than 65 years old, but relatively younger diabetic patients (<65 years) had not been affected by sulfonylurea therapy. And this study indicated that gliclazide could reduce risk of CRC in patients with T2DM, while glimepiride and other sorts of sulfonylurea antidiabetic drugs might increase risk of CRC reversely [118]. Moreover, one meta-analysis also revealed that sulfonylurea was correlated with higher risk of CRC [108]; meanwhile, the other meta-analysis failed to demonstrate the statistically significant correlations between sulfonylurea and risk of CRC in diabetic patients [109]. In addition to sulfonylurea, *α*-glucosidase inhibitor is another crucial oral antidiabetic drug which can control postprandial blood glucose by inhibiting absorption of carbohydrate in the intestine. One cohort study including 134384 patients with T2DM found that acarbose (representative drug of *α*-glucosidase inhibitor) could play a role in protecting from CRC in these patients [119]. At present, epidemiological studies concerning relationship between sulfonylurea, *α*-glucosidase inhibitor, and risk of CRC are still far from satisfactory and need to be discussed further.

## 6. Conclusions

CRC and T2DM have several common risk factors including western diet, obesity, and sedentary lifestyle. Epidemiological studies have identified T2DM as a risk factor for CRC. Underlying biological links between T2DM and colorectal cancer include hyperinsulinemia and IGFs axis, hyperglycemia, adipose tissue dysfunction-induced inflammation, gastrointestinal motility disorder, and impaired immunological surveillance. Underlying diabetes is also associated with worse prognosis of patients with CRC. In this review, we highlight two novel types of anticancer agents for CRC, including inhibitors of IGF-1R and RAGE. Both IGF-1R and RAGE play a crucial role in T2DM-induced colorectal tumorigenesis. IGF-1R- and RAGE-targeted therapies are expected to be ideal therapeutic choices for CRC patients with T2DM in future. Appropriate choice of hypoglycemic therapy regimen, which can reduce the risk of CRC in diabetic patients and improve the prognosis of patients with coexisting diabetes and CRC, is an unmet clinical issue. Through integrating current clinical studies with respect to links between commonly used hypoglycemic agents and CRC, this review was aimed at providing a theoretical basis and clinical evidence for rational use of hypoglycemic drugs. Moreover, regular colonoscopy and surveillance for tumor markers are recommended for patients diagnosed with T2DM for early detection and treatment for CRC.

## Figures and Tables

**Table 1:** Summary of representative association studies between type 2 diabetes mellitus (T2DM) and CRC.

Influence of T2DM on CRC	Ref.	Study design	Main findings
Increased incidence of CRC	Pang et al. [4]	Cohort study (*n* = 512713)	Diabetic patients showed an increased risk of CRC (HR: 1.18; 95% CI: 1.04–1.39).
	Ma et al. [5]	Cohort study (*n* = 134763)	Diabetic patients showed an increased risk of CRC (HR: 1.42; 95% CI: 1.12–1.81).
	Guraya et al. [6]	Meta-analysis (*n* = 924632)	Diabetic patients showed an increased risk of CRC (HR: 1.21; 95% CI: 1.02-1.42).
	Larsson et al. [7]	Cohort study (*n* = 48850)	Diabetic male patients showed an increased risk of CRC (HR: 1.49; 95% CI: 1.14–1.96).
	Campbell et al. [8]	Cohort study (*n* = 154975)	Diabetic male patients showed an increased risk of CRC (RR: 1.22; 95% CI: 1.08-1.44), whereas diabetic female patients failed to show increased risk of CRC (RR: 1.01; 95% CI: 0.82-1.23).
	Jiang et al. [9]	Meta-analysis (*n* = 11692232)	Diabetic patients showed an increased risk of CRC (summary relative risks: 1.27; 95% CI: 1.21-1.34).
Worse prognosis of CRC	Mills et al. [10]	Meta-analysis (*n* = 212888)	CRC patients with T2DM had higher all-cause mortality (RR: 1.17; 95% CI: 1.09-1.25) and cancer-specific mortality (RR: 1.12; 95% CI: 1.01-1.24).
	Dehal et al. [11]	Cohort study (*n* = 393)	CRC patients with T2DM had higher overall mortality (RR: 1.53; 95% CI: 1.28–1.83).
	Huang et al. [12]	Cohort study (*n* = 469)	CRC patients with T2DM had higher overall mortality (RR: 1.21; 95% CI: 1.04–1.41) and CRC-specific mortality (RR: 1.21; 95% CI: 1.02–1.43).
	Barone et al. [13]	Meta-analysis (*n* = 54,740)	CRC patients with T2DM had higher overall mortality (RR: 1.32; 95% CI: 1.24–1.41).
	Stein et al. [14]	Meta-analysis (*n* = 8984)	CRC patients with T2DM had higher overall mortality (RR: 1.32; 95% CI: 1.24–1.41).
	Jeon et al. [15]	Cohort study (*n* = 4131)	Colon cancer patients with T2DM had worse disease-free survival (HR: 1.46; 95% CI: 1.11-1.92) and DFS (HR: 1.45; 95% CI: 1.15-1.84), but such effects were not observed in rectal cancer patients with T2DM.

**Table 2:** Summary of representative anticancer agents targeting IGF-1R and RAGE against CRC.

Agent categories	References	Design	Main findings
Anticancer agents targeting IGF-1R	Leiphrakpam et al. [82]	Preclinical study	The monoclonal anti-IGF1R antibody MK-0646 and IGF-1R tyrosine kinase inhibitor OSI-906 could induce apoptosis of colon cancer cells in vitro and inhibit the growth of subcutaneous CRC xenograft.
	Cohen et al. [83]	Preclinical study	The monoclonal anti-IGF1R antibody CP751,871 could restrain the growth of CRC xenograft and enhance the anticancer efficacy of chemotherapeutic agents including Adriamycin, 5-fluorouracil, or tamoxifen in CRC models.
	Flanigan et al. [84]	Preclinical study	The IGF-1R/insulin receptor tyrosine kinase inhibitor PQIP could improve anticancer efficacy of chemotherapeutic drugs (including oxaliplatin, irinotecan, and 5-fluorocrail) against CRC xenografts.
	Becerra et al. [85]	Clinical study (*n* = 168)	None of the 168 patients with metastatic CRC achieved objective partial or complete response and obtain survival benefits after receiving intravenous therapy of CP751,871.
	Lin et al. [61]	Clinical study (*n* = 64)	Few patients with chemotherapy-refractory colorectal cancer could benefit from treatment with the monoclonal anti-IGF1R antibody SCH71745.
	Sclafani et al. [86]	Clinical study (*n* = 344)	Patients with metastatic CRC failed to gain survival benefits after adding monoclonal anti-IGF1R antibody MK-0646 to irinotecan and cetuximab.
	Cohen et al. [87]	Clinical study (*n* = 64)	The monotherapy of monoclonal anti-IGF1R antibody IMC-A12 failed to exhibit anticancer efficacy for patients with metastatic CRC. The combination of IMC-A12 with epidermal growth factor receptor inhibitor cetuximab did not show additional anticancer activity either.
	Cohn et al. [88]	Clinical study (*n* = 155)	Few patients with metastatic CRC refractory to fluoropyrimidine and oxaliplatin-based chemotherapy gained survival benefits from combination of monoclonal anti-IGF1R antibody AMG 479 and FOLFIRI chemotherapy.
Anticancer agents targeting RAGE	Arabiyat et al. [90]	Preclinical study	Fluoroquinolones could inhibit AGEs and exhibited cytotoxicity against multiple CRC cell lines in vitro.
	Zhang et al. [91]	Preclinical study	Flavonoids and polyphenolic acids extracted from Castanea mollissina Blume showed stronger inhibitory effects on AGEs and cytotoxic activity against CRC cell lines.
	Hafsa et al. [92]	Preclinical study	The extract from Carpobrotus edulis could inhibit AGEs and significantly decrease the CRC cell viability.
	Zhang et al. [94], 2017	Preclinical study	The anti-S100A9 antibody could suppress proliferation and inflammatory response of CRC cells in mice.
	Gnanasekar et al. [95]	Preclinical study	The short hairpin RNA targeting HMGB1 can inhibit proliferation and migration of CRC cells.
	Kuniyasu et al. [44]	Preclinical study	The HMGB1 antisense S-oligodeoxynucleotides could significantly repress growth and invasion of CRC cell lines.

**Table 3:** Nonexhaustive summary of associations between the use of hypoglycemic medications (insulin, insulin analogs, metformin, thiazolidinediones, sulfonylurea, and *α*-glucosidase inhibitor) and the risk of CRC.

Agent categories	Ref.	Design	Main findings
Insulin and its analogs	Ma et al. [96]	Cohort study (*n* = 14916)	C-peptide levels were associated with risk of CRC (RR: 2.7; 95% CI: 1.2-6.2).
	Wei et al. [97]	Nested case-control study (*n* = 32826)	C-peptide levels were associated with risk of colon cancer (RR: 1.76; 95% CI: 0.85-3.63).
	Bu et al. [98]	Meta-analysis (*n* = 491384)	Insulin therapy could increase the risk of CRC. Specifically, insulin use was associated with a statistically significant 115% higher risk of CRC among case-control studies (RR: 2.15; 95% CI: 1.41-3.26), but not among cohort studies (RR: 1.25; 95% CI: 0.95-1.65).
	Wang et al. [99]	Meta-analysis (*n* = 246181)	Insulin use could contribute to the risk of CRC (RR: 1.61; 95% CI: 1.18-1.35).
	Yin et al. [100]	Meta-analysis (*n* = 737562)	Insulin use was significantly associated with risk of CRC (RR: 1.69; 95% CI: 1.25-2.27).
	Chen et al. [101]	Meta-analysis (*n* = 66 324)	Insulin use was associated with an increased risk of CRC (RR: 1.86; 95% CI: 1.58-0-2.19).
	Yang et al. [102]	Cohort study (*n* = 24918)	Insulin therapy significantly increased the risk of CRC (RR: 2.1; 95% CI: 1.2-3.4).
	Wu et al. [103]	Meta-analysis (*n* = 1223812)	Use of insulin analogues (insulin glargine and detemir) was not associated with risk of CRC.
	Pradhan et al. [104]	Cohort study (*n* = 10,734)	Use of long-acting insulin analogs was not associated with an increased risk of colorectal cancer (HR: 0.96; 95% CI: 0.70-1.34).
	But et al. [105]	Cohort study (*n* = 21390)	Use of insulin glargine and insulin detemir was associated with risk of CRC (RR: 1.54; 95% CI: 1.06-2.25).
Metformin	Zhang et al. [107]	Meta-analysis (*n* = 108161)	Metformin use was associated with lower risk of CRC (RR: 0.63; 95% CI: 0.50-0.79).
	Singh et al. [108]	Meta-analysis (*n* = 840787)	Metformin use was associated with an 11% reduction in CRC (RR: 0.89; 95% CI: 0.81-0.99).
	Soranna et al. [109]	Meta-analysis (*n* = 37632)	Metformin use was associated with significantly decreased risk of CRC (RR: 0.64; 95% CI: 0.54-0.76).
	Lee et al. [110]	Cohort study (*n* = 800000)	Metformin could reduce the incidence of CRC (RR: 0.36; 95% CI: 0.13-0.98).
	Lee et al. [111]	Cohort study (*n* = 595)	Metformin use was associated with lower risk of overall mortality (HR: 0.66; 95% CI: 0.476-0.923) and CRC-specific mortality (HR: 0.66; 95% CI: 0.45-0.975) in CRC patients with T2DM.
	Baglia et al. [112]	Cohort study (*n* = 890)	Use of metformin was associated with better overall survival among CRC patients with T2DM (HR: 0.55; 95% CI: 0.34-0.88).
Thiazolidinedione	Singh et al. [108]	Meta-analysis (*n* = 840787)	TZD use was not associated with CRC risk (OR: 0.96; 95% CI: 0.87-1.05).
	Colmers et al. [113]	Meta-analysis (*n* = 2500000)	Use of TZDs was associated with decreased risk of CRC (RR: 0.93; 95% CI: 0.87-1.00).
	Chang et al. [114]	Case-control study (*n* = 606,583)	Rosiglitazone was associated with reduced risk (OR: 0.86; 95% CI: 0.76-0.96), but such protective benefits has not seen in pioglitazone.
	Chen et al. [115]	Case-control study (*n* = 24,496)	TZD use was associated with reduced CRC risk (OR: 0.86; 95% CI: 0.79-0.94).
	Liu et al. [116]	Meta-analysis (*n* = 2470768)	TZD use was associated with reduced CRC risk (RR: 0.91; 95% CI: 0.84-0.99).
	Govindarajan et al. [117]	Cohort study (*n* = 87678)	The TZD-associated risk reduction for CRC did not reach statistical significance.
Sulfonylurea	Singh et al. [108]	Meta-analysis (*n* = 840787)	Sulfonylurea use was not associated with risk of CRC (OR: 1.11; 95% CI: 0.97-1.26).
	Soranna et al. [109]	Meta-analysis (*n* = 37632)	Sulfonylurea use was not associated with risk of CRC.
	Shin et al. [118]	Nested case-control study (*n* = 8436)	Glimepiride use increased the risk for CRC (RR: 1.14; 95% CI: 1.06-1.22), whereas gliclazide decreased the risk for CRC (RR: 0.85; 95% CI: 0.72-1.00).
*α*-Glucosidase inhibitor	Tseng et al. [119]	Cohort study (*n* = 1343484)	Acarbose use reduced the risk of CRC in patients with T2DM in a dose-dependent manner (HR: 0.66; 95% CI: 0.59–0.74).
